# Determining an Appropriate Sample Size for Qualitative Interviews to Achieve True and Near Code Saturation: Secondary Analysis of Data

**DOI:** 10.2196/52998

**Published:** 2024-07-09

**Authors:** Claudia M Squire, Kristen C Giombi, Douglas J Rupert, Jacqueline Amoozegar, Peyton Williams

**Affiliations:** 1 RTI International Research Triangle Park, NC United States

**Keywords:** saturation, sample size, web-based data collection, semistructured interviews, qualitative, research methods, research methodology, data collection, coding, interviews, interviewing, in-depth

## Abstract

**Background:**

In-depth interviews are a common method of qualitative data collection, providing rich data on individuals’ perceptions and behaviors that would be challenging to collect with quantitative methods. Researchers typically need to decide on sample size a priori. Although studies have assessed when saturation has been achieved, there is no agreement on the minimum number of interviews needed to achieve saturation. To date, most research on saturation has been based on in-person data collection. During the COVID-19 pandemic, web-based data collection became increasingly common, as traditional in-person data collection was possible. Researchers continue to use web-based data collection methods post the COVID-19 emergency, making it important to assess whether findings around saturation differ for in-person versus web-based interviews.

**Objective:**

We aimed to identify the number of web-based interviews needed to achieve true code saturation or near code saturation.

**Methods:**

The analyses for this study were based on data from 5 Food and Drug Administration–funded studies conducted through web-based platforms with patients with underlying medical conditions or with health care providers who provide primary or specialty care to patients. We extracted code- and interview-specific data and examined the data summaries to determine when true saturation or near saturation was reached.

**Results:**

The sample size used in the 5 studies ranged from 30 to 70 interviews. True saturation was reached after 91% to 100% (n=30-67) of planned interviews, whereas near saturation was reached after 33% to 60% (n=15-23) of planned interviews. Studies that relied heavily on deductive coding and studies that had a more structured interview guide reached both true saturation and near saturation sooner. We also examined the types of codes applied after near saturation had been reached. In 4 of the 5 studies, most of these codes represented previously established core concepts or themes. Codes representing newly identified concepts, other or miscellaneous responses (eg, “in general”), uncertainty or confusion (eg, “don’t know”), or categorization for analysis (eg, correct as compared with incorrect) were less commonly applied after near saturation had been reached.

**Conclusions:**

This study provides support that near saturation may be a sufficient measure to target and that conducting additional interviews after that point may result in diminishing returns. Factors to consider in determining how many interviews to conduct include the structure and type of questions included in the interview guide, the coding structure, and the population under study. Studies with less structured interview guides, studies that rely heavily on inductive coding and analytic techniques, and studies that include populations that may be less knowledgeable about the topics discussed may require a larger sample size to reach an acceptable level of saturation. Our findings also build on previous studies looking at saturation for in-person data collection conducted at a small number of sites.

## Introduction

### Background

In-depth interviews are commonly used to collect qualitative data for a wide variety of research purposes across many subject matter areas. These types of interviews are an ideal approach for examining individuals’ perceptions and behaviors at a level of depth, complexity, and richness that would be challenging to achieve with quantitative data collection methods. Typically, trained interviewers conduct interviews using a guide designed to address the study’s key research aims by asking a series of questions and probes ordered by topic. These interview guides can range from highly structured to completely unstructured (eg, loosely organized conversations). Following the completion of data collection, interview notes and transcripts generated from audio recordings of the interviews are analyzed to assess for patterns in responses among the interviewees or subsets of the participants [1,2].

During the COVID-19 pandemic, web-based data collection became increasingly common, as traditional in-person data collection was not possible, and researchers continue to use web-based data collection methods post the COVID-19 emergency, citing advantages such as accessing marginalized populations, achieving greater geographic diversity, being able to offer a more flexible schedule, and saving on travel expenses [3]. Potential concerns about web-based data collection, such as the inability to build rapport and data richness, have been largely unfounded [3,4].

While we do not expect web-based data collection to supplant in-person research, it continues to show signs of growth. To date, much of the research on qualitative methods has focused on in-person data collection. Consequently, it will be important to conduct research to determine if previous widely accepted findings hold true for web-based data collection.

Researchers typically make a priori decisions about the number of interviews to conduct with the aim of balancing the need for sufficient data with resource limitations and respondent burden. The concept of saturation is frequently used to justify the study’s rigor with respect to the selected sample size. To provide empirically based recommendations on adequate minimum sample sizes, researchers have conducted studies to assess when saturation occurs. However, multiple types of saturation exist—such as theoretical, thematic, code, and meaning—and within each type of saturation, the definitions and measurement approaches used by investigators vary substantially, as does the level of detail researchers report in publications about their methods for achieving and assessing saturation [5].

This study aimed to examine the number of interviews needed to obtain code saturation for 5 recently conducted studies funded by the Food and Drug Administration [6] involving web-based interviews. Specifically, how many web-based interviews are needed to obtain true code saturation (ie, the use of 100% of all codes applied in the study) and how many web-based interviews are needed to achieve near code saturation (ie, the use of 90% of all codes applied in the study)?

### Literature Review

Multiple authors have defined saturation as the point during data collection and analysis, at which no new additional data are found that reveal a new conceptual category [7-13] or theme related to the research question—an indicator that further data collection is redundant [11]. Additionally, Coenen et al [14] specified that no new second-level themes are revealed in 2 consecutive focus groups or interviews.

Other authors have distinguished between various types of saturation. One of the most common types of saturation mentioned in the literature is theoretical saturation, which emerges from grounded theory and occurs when the concepts of a theory are fully reflected in the data and no new insights, themes, or issues are identified from the data [5,11,12,15-18]. Hennink et al [17] expanded this definition, adding that all relevant conceptual categories should have been identified, thus emphasizing the importance of sample adequacy over sample size. Guest et al [15] operationalized the concept of theoretical saturation as the point in data collection and analysis when new information produces little or no change to the codebook, and van Rijnsoever [19] operationalized it as being when all the codes have been observed once in the sample.

Some authors have defined theoretical saturation, thematic saturation, and data saturation as the same concept [16,18], whereas others have defined these terms differently [12,20]. For example, some authors have defined thematic saturation as the point where no new codes or themes are emerging from the data [12,21]. For thematic saturation to be achieved, data should be collected until nothing new is generated [20,22]. Data saturation has been defined as the level to which new data are repetitive of the data that have been collected [12,23,24].

Furthermore, Hennink et al [17] distinguished between code saturation and meaning saturation. Code saturation is based on primary or parent codes and relates to the quantity of the data (“hearing it all”). Meaning saturation is based on sub or child codes and relates to the quality or richness of the data (“understanding it all”). Constantinou et al [7] made the point that it is the categorization of the raw data, rather than the data, that are saturated.

The literature reflects multiple methods that have been used to determine saturation [7-10,13-18,21,25]. Sim et al [26] discussed the four general approaches that have been used to determine sample size for qualitative research: (1) rules of thumb, based on a combination of methodological considerations and past experience; (2) conceptual models, based on specific characteristics of the proposed study; (3) numerical guidelines derived from the empirical investigation; and (4) statistical approaches, based on the probability of obtaining a sufficient sample size.

For example, Galvin [9] used a statistical approach based on binomial logic to establish the relationship between identifying a theme in a particular sample and within the larger population; for example, number of chances of detecting a theme if that theme exists within number of the population. Using the probability equation, the researcher can determine the number of interviews needed for a stated level of confidence that all relevant themes held by a certain proportion of the population will occur within the interview sample. This method assumes the researcher knows in advance the emergent themes from the study and at what rate they may occur.

Constantinou et al [7] used the comparative method for themes saturation, which relies on both a deductive and an inductive approach to generate codes (keywords extracted from the participants’ words) and themes (codes that fall into similar categories). Themes are compared across interviews, and theme saturation is reached when the next interview does not produce any new themes. The sequence of interviews is reordered multiple times to check for order-induced error. When exploring the various methods for determining saturation, researchers reached different conclusions on when saturation was achieved (findings on saturation by other authors are present in Multimedia Appendix 1) [7-10,13-17,21,25,27,28].

Most studies assessing saturation focused on in-person data collection or did not specify the data collection method. Given recent increases in web-based data collection, studies assessing saturation for web-based interviews are critical to ensure that recommendations regarding sample size are tailored to the mode of data collection [4]. While there is evidence to suggest that the content of data coded from in-person as compared with web-based interviews is conceptually similar [29], this is a relatively new area of exploration. Rapport may be higher with in-person as compared with web-based interviews [30], which may impact the amount and type of content generated. Additionally, participants in web-based data collection studies are more geographically diverse and may be more likely to be non-White, less educated, and less healthy than participants in in-person data collection studies [31].

## Methods

### Study Design

This study was based on analyses from data collected for 5 Food and Drug Administration–funded studies conducted using web-based platforms, such as Zoom (Zoom Video Communications) and Adobe Connect (Adobe Systems), and focused on patients with underlying medical conditions or on health care providers who provide primary or specialty care to patients. All platforms used for these interviews offered audio and video components and allowed for the sharing of stimuli on screen. A brief description of each study is provided in Table 1. Each study’s data had been coded and stored using NVivo software (version 11; QSR International).

**Table 1:** Description of studies included in analysis of code saturation: sample size, eligibility criteria, topics covered, length of interview, number of questions, and regions and states covered.

Study name	Sample size, n	General eligibility criteria	Primary objectives	Summary of topics	Length of interview (minutes)	Number of interview questions	Regions and states covered
Study A	30	Patients diagnosed with a condition treated by biologic medications (eg, cancer, inflammatory bowel disease, and diabetes)	Obtain feedback on multimedia educational materials about biosimilar biologic medications	Biosimilar awareness Feedback on educational materials (eg, comprehension, main message, and format) Behavioral intentions	90	37 main questions Questions identified as high, average, and low priority	Regions: Northeast, Midwest, South, and West States: 14
Study B	48	Patients diagnosed with vulvovaginal atrophy or type 2 diabetes	Explore how patients use boxed warnings when making decisions about prescription drugs and how well the warnings meet patients’ information needs	Prescription drug information needs Boxed warning awareness, interpretation, and perceptions Behavioral intentions	30	13 main questions	Regions: Northeast, Midwest, South, and West States: not available
Study C	70	Primary care physicians or specialists who write at least 50 prescriptions per week	Assess how primary care physicians and specialists access, understand, and use prescription drug labeling information, including information on labels for drugs that have multiple indications.	Resources to find information about prescription drugs Background on prescribing information Interpretation of language in the prescribing information	60	36 main questions	Regions: Northeast, Midwest, South, and West States: 26
Study D	35	Patients diagnosed with type 2 diabetes	Understand how patients weigh the potential benefits against possible risks and side effects, dosage and administration characteristics, and costs when selecting treatments for chronic health conditions.	Background information on condition Treatment decisions and discussion of attributes Ranking attributes Condition-specific statements about attributes Market claims	60	20 main questions	Regions: Northeast, Midwest, South, and West States: 9
Study E	35	Patients diagnosed with psoriasis	Understand how patients weigh the potential benefits against possible risks and side effects, dosage and administration characteristics, and costs when selecting treatments for chronic health conditions.	Background information on condition Treatment decisions and discussion of attributes Ranking attributes Condition-specific statements about attributes Market claims	60	21 main questions	Regions: Northeast, Midwest, South, and West States: 9

### Ethical Considerations

This project was determined to not research with human participants by Research Triangle Institute’s institutional review board (STUDY00021985). The original 5 studies that this project is based on were reviewed by Research Triangle Institute’s institutional review board and were determined to be exempt under category 2ii. Participants in these studies were provided information about measures used to protect their privacy and the confidentiality of their data in the study’s consent forms. All participants were provided compensation for their time (the amount and type varied by study).

### Data Preparation and Analysis

We established and applied a systematic approach to analyze all 5 study data sets. Our analytic approach was organized into 2 stages—data preparation and data analysis.

#### Data Preparation

First, because previous interviews sometimes influence moderator probes—for example, the moderator asks a follow-up question based on something they heard in a previous interview—we sorted interviews from each study by interview order. We then extracted code- and interview-specific data from the NVivo databases—including transcript name, code name, number of files coded, number of associated parent and child codes, and number of coding references—and compiled these data in an Excel (Microsoft Corp) file. We then updated the Excel file with important code and interview characteristics, including the order in which interviews were conducted, whether each code was directly (ie, child codes) or indirectly (ie, parent codes) applied to transcripts (in a tiered coding scheme, direct codes are those that have no child codes, whereas indirect codes function as “parents” that have additional codes nested beneath them), and the point at which each code was first applied to an interview. Finally, we created pivot tables within each Excel file to compile the data.

#### Data Analysis

Once the data were compiled, the data summaries were examined to determine when true saturation and near saturation occurred during data collection. True saturation was defined as 100% of all applied codes being used; near saturation was defined as 90% of all applied codes being used. We calculated saturation separately for each study’s data set, and we calculated saturation separately for all codes (ie, parent and child codes) as compared with direct codes (ie, child codes only). True saturation and near saturation points were identified by calculating the cumulative percentage of new codes for each interview, flagging when 100% and 90% of applied codes had been used.

## Results

### True and Near Saturation

**Figure 1:**
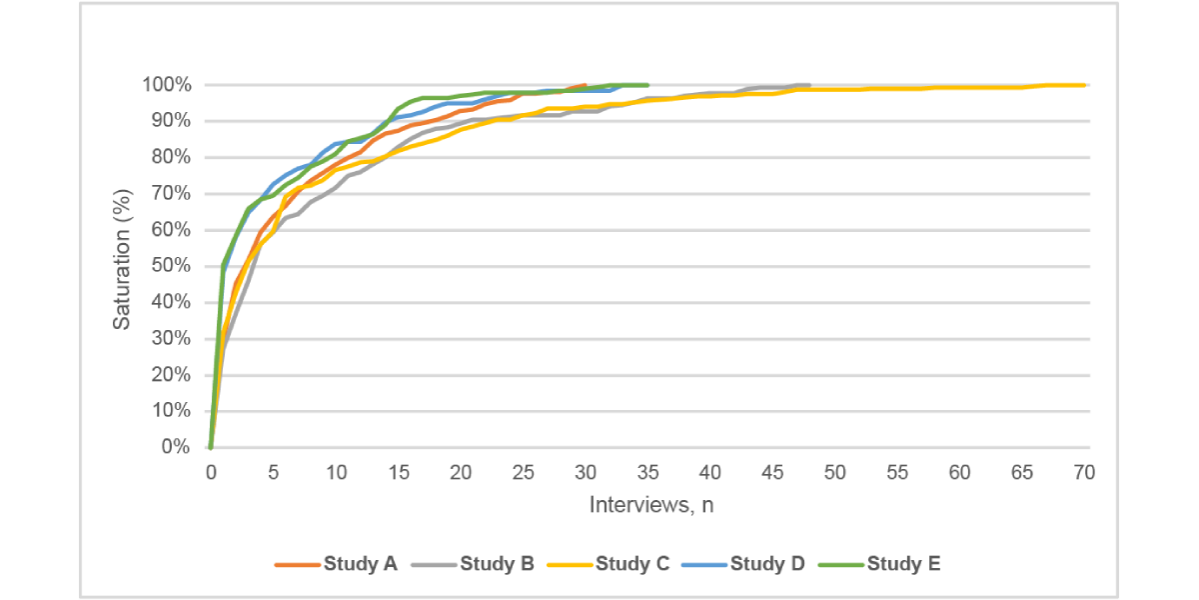
Illustration of cumulative percentage of new codes applied by study to reach true and near saturation.

The number of web-based interviews used across the 5 studies ranged from 30 to 70 (Table 2). True saturation (100% use of all applied codes) was reached in the final or near final interview (Figure 1), suggesting that, even with a large sample size, additional interviews are likely to continue uncovering a small number of new codes or findings.

**Table 2:** Interviews needed to reach true and near saturation by study.

Study	Total interviews, n	Coding: total codes in codebook, n	True saturation: interviews needed, n (%)	Near saturation: interviews needed, n (%)
Study A	30	657	30 (100)	18 (60)
Study B	48	313	47 (98)	21 (44)
Study C	70	362	67 (96)	23 (33)
Study D	35	205	33 (94)	15 (43)
Study E	35	200	32 (91)	15 (43)

Across all studies, near saturation (90% use of all applied codes) was reached near—and often before—the midpoint of data collection. In other words, only a small number of new codes or findings were uncovered once the first half of the sample had been interviewed. In terms of absolute numbers, the point at which near saturation was reached occurred between 33% and 60% (n=15-23) of planned interviews (Table 2). Despite the participants being more geographically, and possibly demographically, diverse compared with typical in-person participants, our findings were similar to previous studies on saturation [10,15,17].

We examined the types of codes applied after near saturation had been reached. In 4 of the 5 studies, most of these codes (n=8-33, 57%-62%) represented previously established core concepts or themes, such as a trusted source of information, a behavioral intention, or a recommended change to educational material. Codes representing newly identified concepts (n=2-8, 10%-15%), other miscellaneous responses (eg, “in general”; n=6-9, 13%-41%), uncertainty or confusion (eg, “don’t know”; n=0-6, 0%-11%), or categorization for analysis (eg, “correct as compared with incorrect”; n=0-3, 0%-4%) were less commonly applied after near saturation had been reached.

The overwhelming majority of codes applied after near saturation (n=9-41, 73%-82%) had already been established in study codebooks before analysis. Only a small number of codes applied after this point (n=4-20, 18%-27%) were conceptually distinct enough to merit updating the study codebooks by including them. Likewise, most of the codes used after near saturation (n=11-35, 44%-64%) were applied to only a single interview. Far fewer codes were applied to 2 interviews (n=0-13, 0%-27%), 3 interviews (n=0-6, 0%-21%), or 4 or more interviews (n=0-12, 0%-21%).

Study B was an outlier in terms of codes applied after near saturation. This study had fewer codes representing core established concepts (n=8, 28%) and more codes representing newly identified concepts (n=7, 24%) or providing categorization for analysis (n=3, 10%) than other studies. The study also had a much higher proportion of new codes (n=20, 69%) that were added to the study codebook during analysis. These differences may be because the study sampled 2 populations with very different medical conditions (ie, type 2 diabetes as compared with vulvovaginal atrophy), leading to a broader range of applied codes.

In examining the relationship between the number of codes in the codebook for each study, the study with the most codes (study A: 657 codes) required the largest number of interviews to reach both true saturation and near saturation. However, this pattern did not hold true for the remainder of the studies. The study with the next highest number of codes (study C: 362 codes) was third to reach true saturation and last to reach near saturation.

### Parent and Child Codes

**Figure 2:**
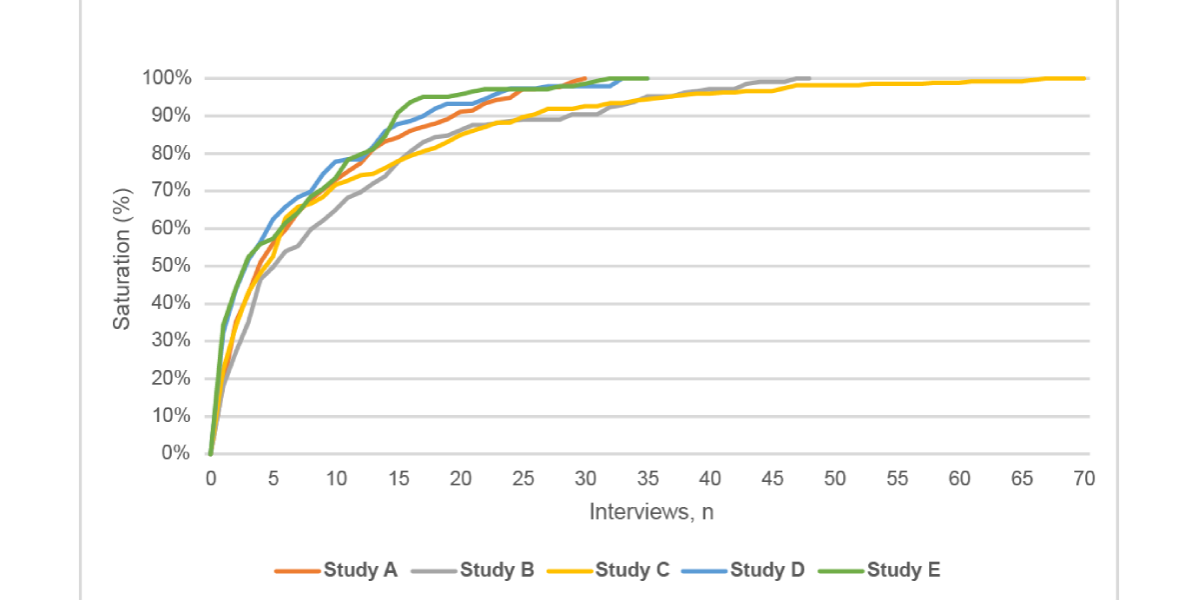
Illustration of cumulative percentage of new codes applied by study to reach true and near saturation (parent codes only).

All 5 study codebooks included both parent (ie, top-level codes) and child codes (ie, subcodes). We examined saturation using two analytic lenses—(1) all codes (parent and child) and (2) parent codes only—to determine if there were differences in when saturation was reached. We found no differences in when true saturation was reached. However, near saturation was reached slightly later (ie, after an additional 3 to 4 interviews) when examining only parent codes (Figure 2).

### Differences by Study

In total, 3 of the studies had codebooks that consisted almost entirely of deductive (ie, concept-driven) codes, whereas the codebooks in the remaining 2 studies contained a mix of both deductive and inductive (ie, data-driven) codes. Although the results were largely consistent across the 5 studies, as expected, the studies that relied heavily on deductive coding reached both true saturation and near saturation sooner. This finding suggests that studies using more inductive coding and analytic techniques may require slightly larger sample sizes to reach saturation.

### Structure of an Interview Guide

Although all the studies used a semistructured interview guide, the level of structure varied across studies. The 3 studies (ie, studies C, D, and E) that had a more structured interview guide (eg, questions for which participants were asked their preference among discrete choices or the range of likely answers was limited) reached both true saturation and near saturation sooner. In fact, the study with the most structured guide reached near saturation the soonest, although it fell in the middle for true saturation. This finding suggests that studies using a less structured interview guide may need to conduct more interviews to reach an acceptable level of saturation.

## Discussion

### Principal Findings

Although true saturation was not reached until the final interview or close to the final interview, near saturation was reached much sooner, ranging from just below to just above the midpoint of data collection, with most of the studies falling just below the midpoint. Although additional interviews conducted after near saturation may result in new information, our findings suggest there may be diminishing returns relative to the resources expended. We have identified several study characteristics that researchers can consider when making decisions on sample size for web-based interviews.

Although our findings were mostly consistent across the 5 studies we examined, near saturation was reached sooner on the studies that consisted of largely deductive codes compared with those that had a greater number of inductive codes. Consequently, researchers should consider their analytic approach when determining sample size. Studies that intend for the coding scheme to be iterative throughout the coding process may want to err on the side of having a slightly higher sample size than if the codebook is expected to consist largely of deductive codes tied to the interview guide.

These studies ranged in length from 30 to 90 minutes, and a majority (n=3) lasted 60 minutes. Although the 90-minute study reached both true saturation and near saturation at the latest point, the shortest interview (at 30 minutes) required the second-highest number of interviews to reach both saturation points. Although the length of the interview may be a minor consideration, the level of structure of the interview guide and the types of codes used seem to be larger drivers.

Our findings point to the need for a slightly higher number of interviews to reach an acceptable level of saturation—categorized by us as near code saturation—than what has been found in other studies. For example, Guest et al [15] found that 6 interviews were enough to get high-level themes, reaching a plateau at 10 to 12 interviews. Similarly, Young and Casey [27] found that near code saturation was reached at 6 to 9 interviews.

Our findings also build on previous studies looking at saturation for in-person data collection conducted at a small number of sites. Data from our studies included participants from all US Census Bureau regions, which provides support that these findings may be more generalizable than previous studies.

### Limitations

Our study had several limitations. First, our analysis was conducted on a sample of 5 studies that had similarities. All the studies were related to the medical field, and our study populations (patients with an identified medical condition and health care providers) were knowledgeable about the topics discussed. Second, all the studies were conducted using semistructured interview guides that leaned toward being more structured (ie, interviewers largely stuck to scripted probes as compared with guides that allow for unscripted follow-up probes and unstructured conversations). Additionally, all the studies used a similar approach to coding by using a mix of both deductive and inductive codes (though to varying extents). Consequently, studies with a less structured approach to both the interview and coding process may yield different results. Finally, all our studies are broadly classified as social science research. The findings for other fields of inquiry, such as economic or medical studies, may differ.

### Conclusions

Saturation is an important consideration in planning and conducting qualitative research, yet, there is no definitive guidance on how to define and measure saturation, particularly for web-based data collection, which allows for data to be collected from a more geographically diverse sample. Our study provides support that near saturation may be a sufficient measure to target and that conducting additional interviews after that point may result in diminishing returns. Factors to consider in determining how many interviews to conduct include the structure and type of questions included in the interview guide, the coding structure, and the population being studied. Studies with less structured interview guides, studies that rely heavily on inductive coding and analytic techniques, and studies that include populations that may be less knowledgeable about the topics discussed may require a larger sample size to reach an acceptable level of saturation. Rather than trying to reach a consensus on the number of interviews needed to achieve saturation in qualitative research overall, we recommend that future research should explore saturation within different types of studies, such as different fields of inquiry, subject matter, and populations being studied. Creating a robust body of knowledge in this area will allow researchers to identify the guidance that best meets the needs of their work.

## Appendix

Multimedia Appendix 1 Achieving saturation in interviews: saturation type, methods for achieving saturation, and findings by other authors.

## Data Availability

The data sets analyzed during this study are available from the corresponding author on reasonable request.
