# Supplementary material for: Determining an Appropriate Sample Size for Qualitative Interviews to Achieve True and Near Code Saturation: Secondary Analysis of Data
Source: J Med Internet Res. 2024 Jul 9;26:e52998. doi: 10.2196/52998 (PMC11267098; doi:10.2196/52998)
Supplement: Multimedia Appendix 1 [file jmir_v26i1e52998_app1.docx]

| Citation | Saturation type | Method for achieving saturation | Finding |
| --- | --- | --- | --- |
| Coenen et al [14] | Data saturation | Compared 2 approaches to conducting focus groups and individual interviews: an open approach and an approach based on ICF^a^ | - Open approach (open-ended questions were used): saturation reached after 9 interviews - ICF-based approach (first level of the ICF classification was added to the open-ended questions): saturation reached after 12 interviews |
| Constantinou et al [7] | Thematic saturation | Used CoMeTS^b^ | - Theme saturation reached at interview 5 - After reordering interviews 3 different ways, theme saturation reached at the seventh and eight interviews |
| Francis et al [8] | Data saturation | Two steps:   - Specified a priori a minimum sample size for initial analysis, which depends on the complexity of the research questions or interview guide, the diversity of the sample, and the nature of the analysis - Specified a priori how many more interviews will be conducted without new ideas emerging (stopping criterion) | - After 10 interviews (the initial analysis sample), 57 shared beliefs identified; no new shared beliefs in interviews 11 or 12; 2 new shared beliefs at interview 13; applying the stopping criterion indicates that study-wise saturation was not achieved in study 1 - Saturation was achieved after 17 interviews in study 2 |
| Fugard and Potts [25] | Thematic saturation | Conducted a statistical calculation of saturation based on expected theme prevalence within the population, number of desired instances of the theme, and desired power of the study | - To have 80% power to detect 2 instances of a theme with 10% prevalence, 29 interviews are required |
| Galvin [9] | Thematic saturation | Used a statistical approach, based on binomial logic, to ascertain the relationship between theme identification in a particular sample and the larger population | - If the researcher needs to be at least 95% confident that all the issues have emerged that are represented in 10% or more of the population, then 29 interviews are required |
| Guest et al [15] | Thematic saturation | Operationalized saturation as a proportion: the number of identified themes at a given point in analysis divided by the total number of themes identified in the entire sample; level of saturation reported as the point at which, post facto, 80% or 90% of themes in a data set are identified | - Saturation reached after 12 interviews; basic elements for meta-themes were present as early as 6 interviews |
| Guest et al [10] | Thematic saturation | Three elements: base size, run length, and new information threshold   - Base size: the minimum number of interviews that should be reviewed or analyzed to calculate the amount of information already gained - Run length: the number of interviews within which we look for and calculate new information; the number of new themes found in the run defines the numerator in the saturation ratio - New information threshold: what level of paucity of new information should we accept as indicative of saturation? | - Reached <5% new information threshold at 6 interviews across all base sizes with a run length of 2 interviews - Reached <5% new information threshold at 7 interviews across all base sizes with a run length of 3 interviews - Reached 0% new information threshold at 11 interviews across all base sizes with a run length of 2 interviews - Reached 0% new information threshold at 14 interviews across all base sizes with a run length of 3 interviews |
| Hagaman and Wutich [16] | Thematic saturation | Used Ryan and Bernard’s [32] repetition approach to identify themes | - Top 3 themes identified at least once in as few as 5 interviews; took more than 24 interviews to elicit the top 3 themes from 3 different respondents - 9 meta-themes identified at least once in as few as 8 interviews; took more than 39 interviews to elicit the 9 meta-themes from 3 different respondents - 16 or fewer interviews needed to identify common themes from a fairly homogenous group |
| Hennink et al [17] | Code saturation; meaning saturation | Compared 2 approaches to assessing saturation: code and meaning saturation | - Code saturation reached at 9 interviews - Meaning saturation reached at 16-24 interviews |
| Turner-Bowker et al [13] | Code saturation | Divided the sample into quartiles and then compared the number of responses elicited from the first 25% of participants to the next 25% of participants, from the first 50% of participants to the next 25% of participants, and then from the first 75% of participants to the last 25% of participants | - 84% of concepts emerged by the 10th interview; 92% emerged by the 15th interview; 97% emerged by the 20th interview; 99% emerged by the 25th interview |
| Weller et al [21] | Thematic saturation; salience | Used a quantitative model to predict the unique number of items contributed by each additional respondent, and saturation was defined as the point where fewer than 1 new item per person would be expected | - The median sample size for reaching saturation was 75 (range 15-194) interviews - Small samples (n=10) produced 95% of the most salient ideas |
| Young and Casey [27] | Code saturation | Used retrospective data from 3 studies; used a random number generator to draw 10 random subsamples of each size from 5 through 10 for individual interviews; examined to see what proportion of the codes and larger themes from each original study’s full sample were present within each subsample | - Near code saturation reached with a sample size of 6-9 interviews - Partial theme representation required 4-6 interviews - Substantial theme completion required 7-20 interviews |

^a^ICF: International Classification of Functioning, Disability and Health.

^b^CoMeTS: comparative method for themes saturation.
